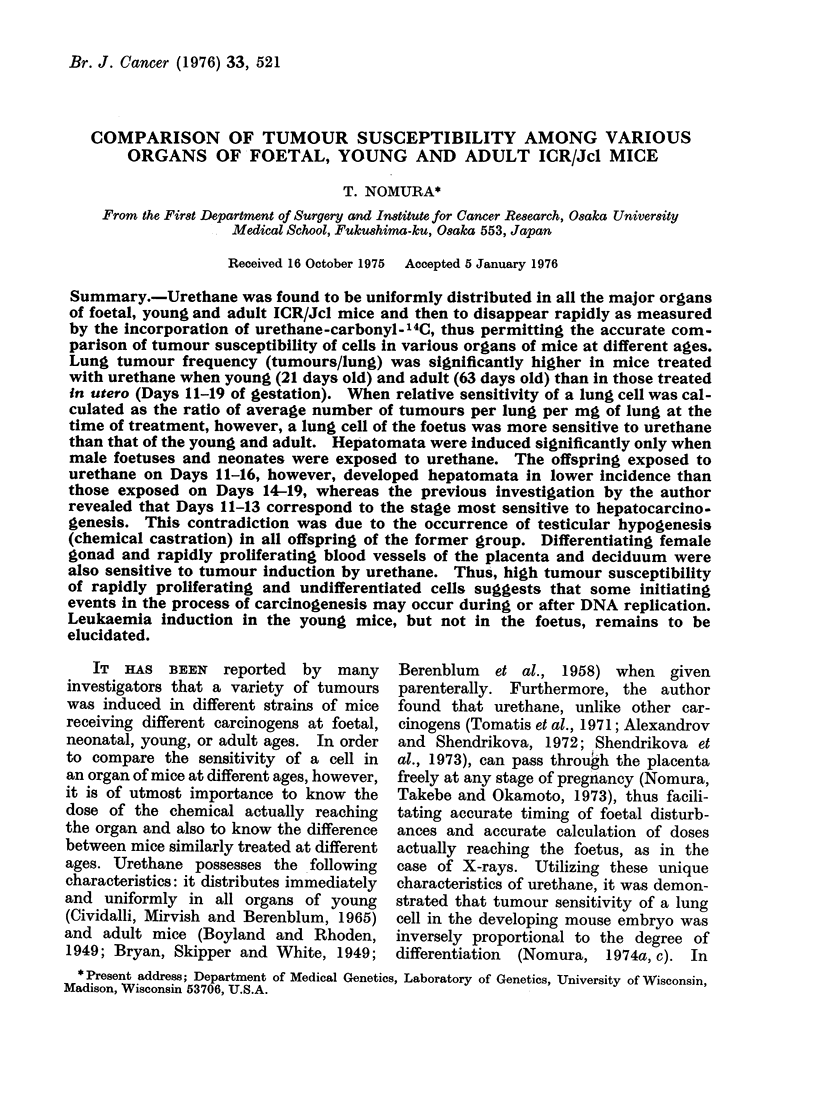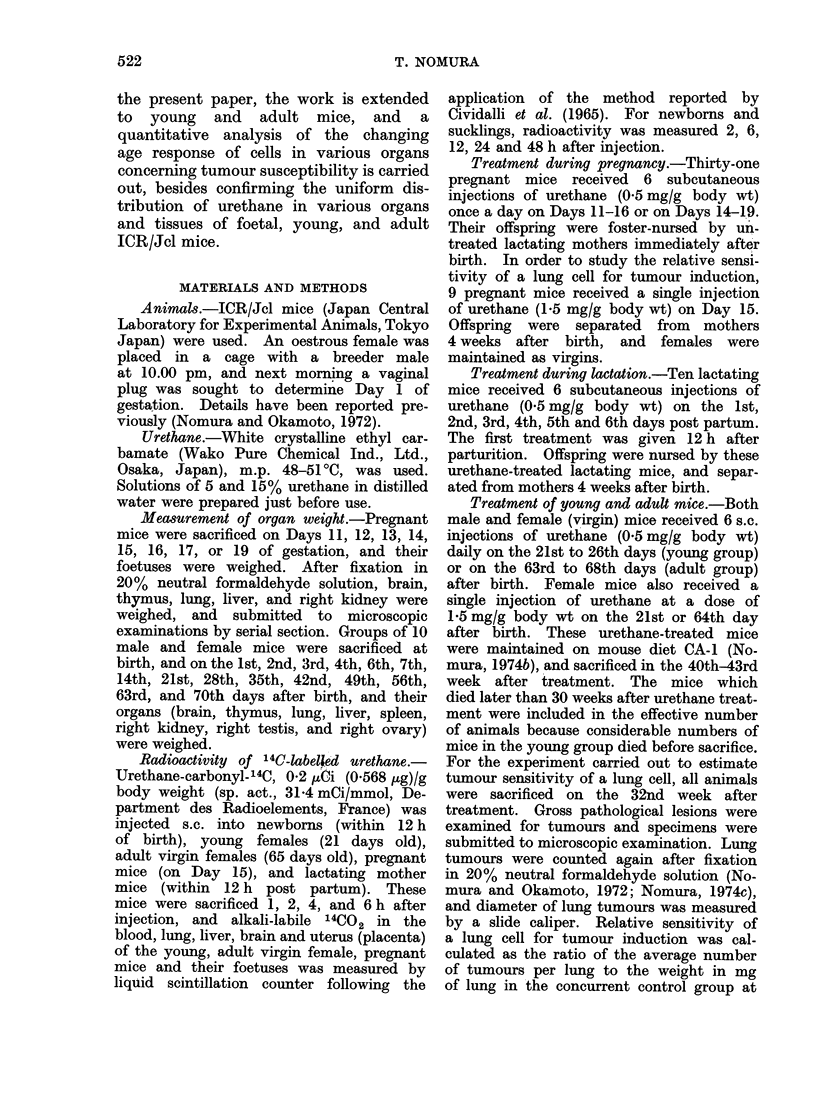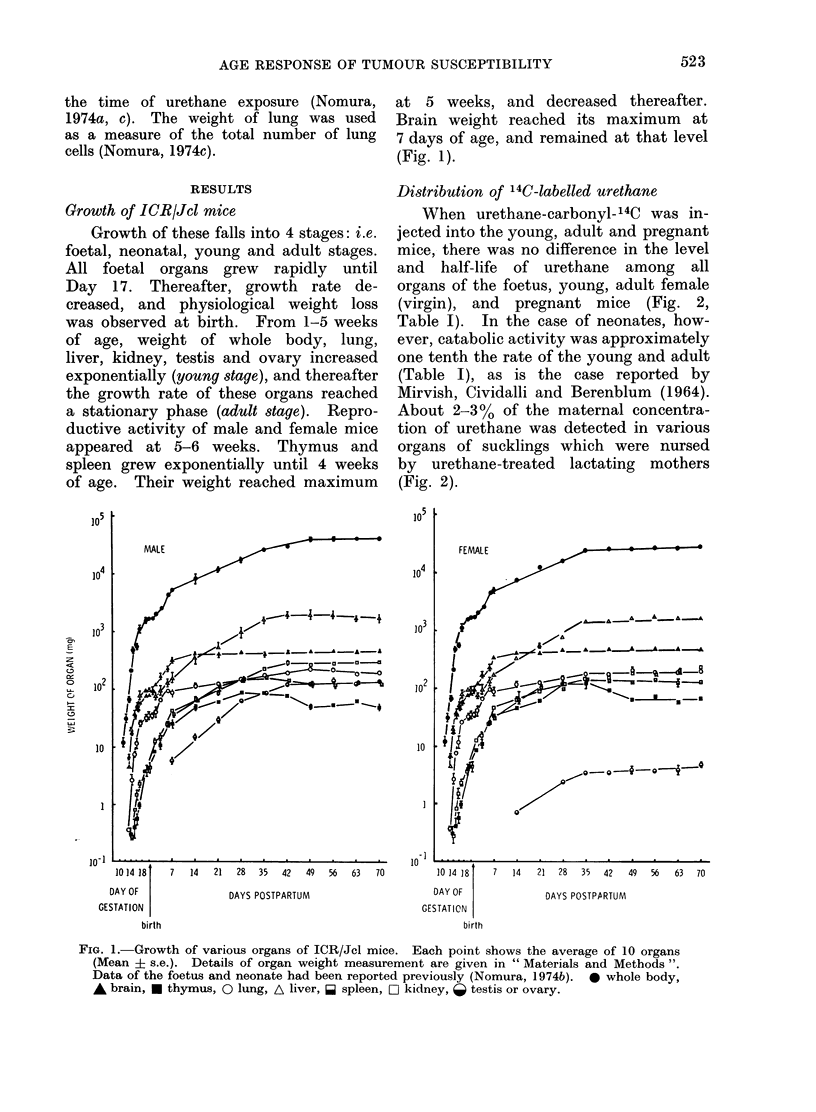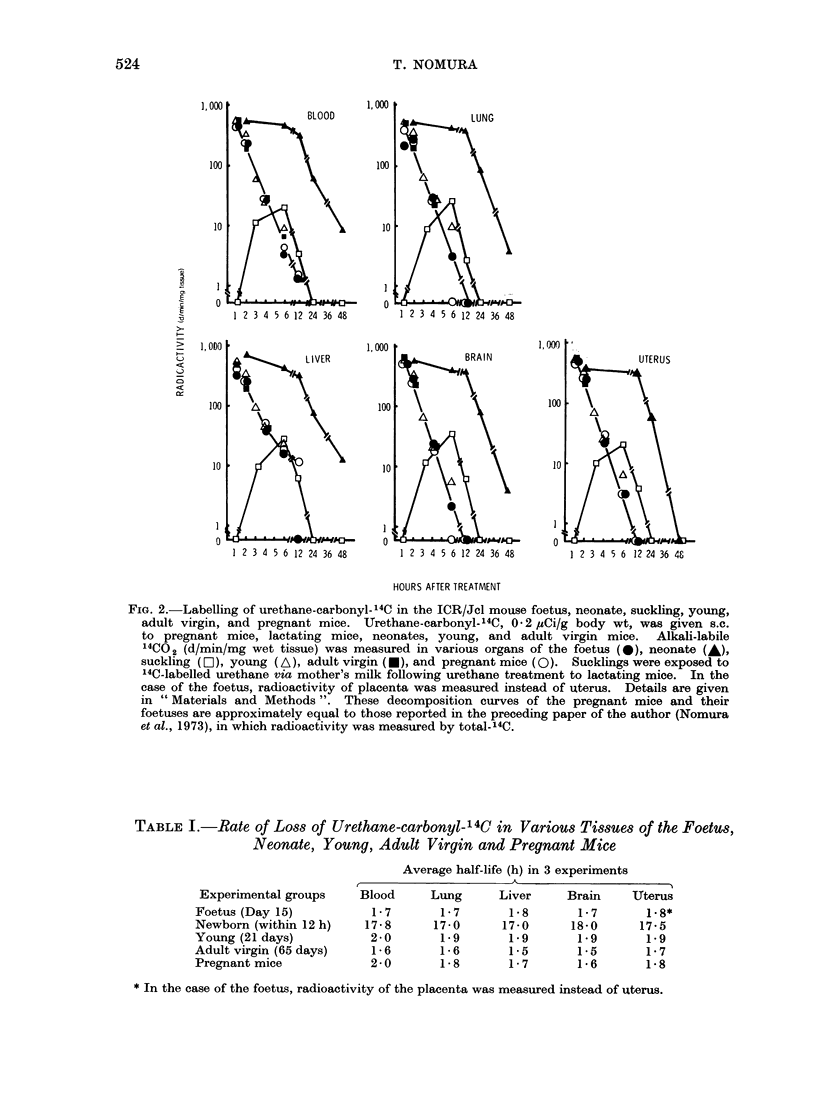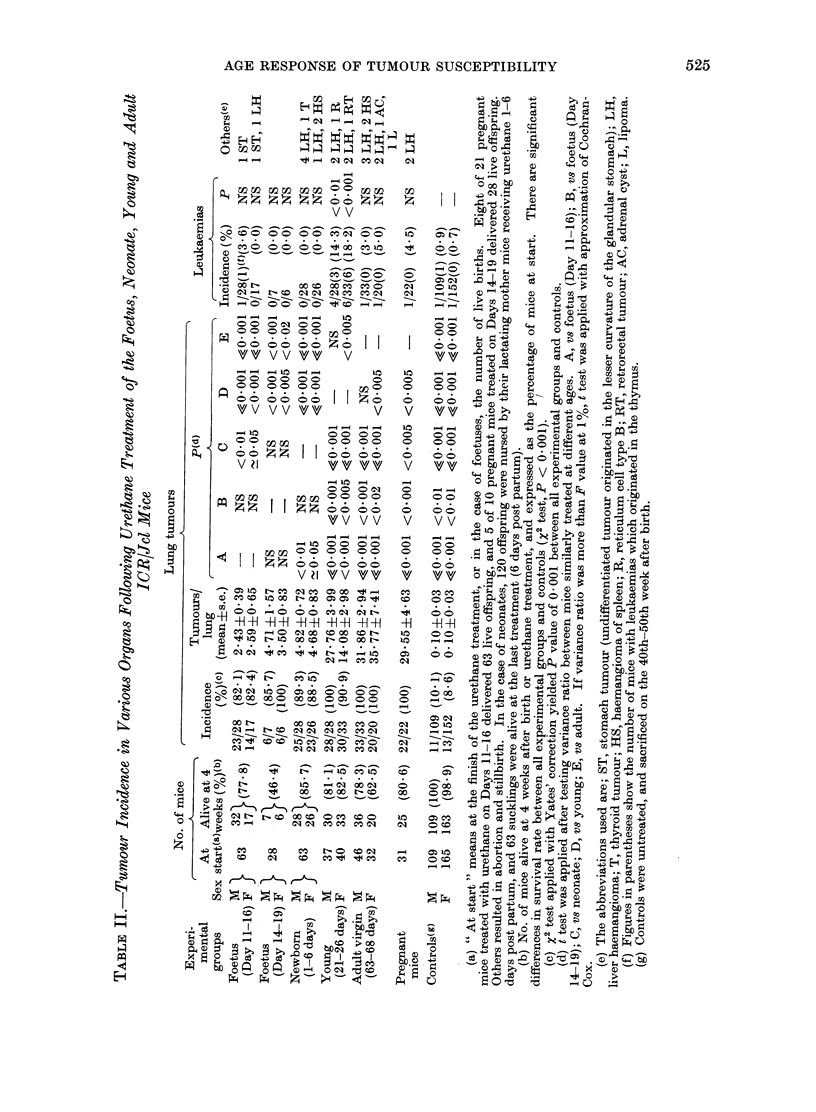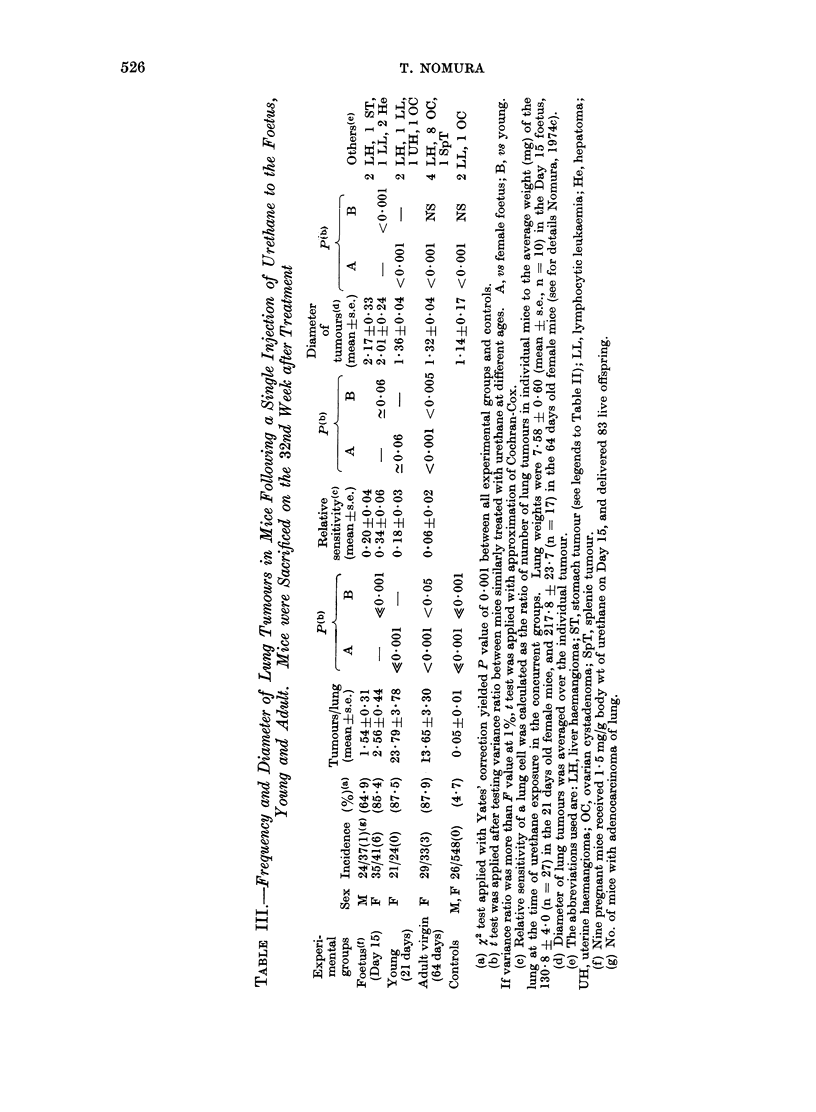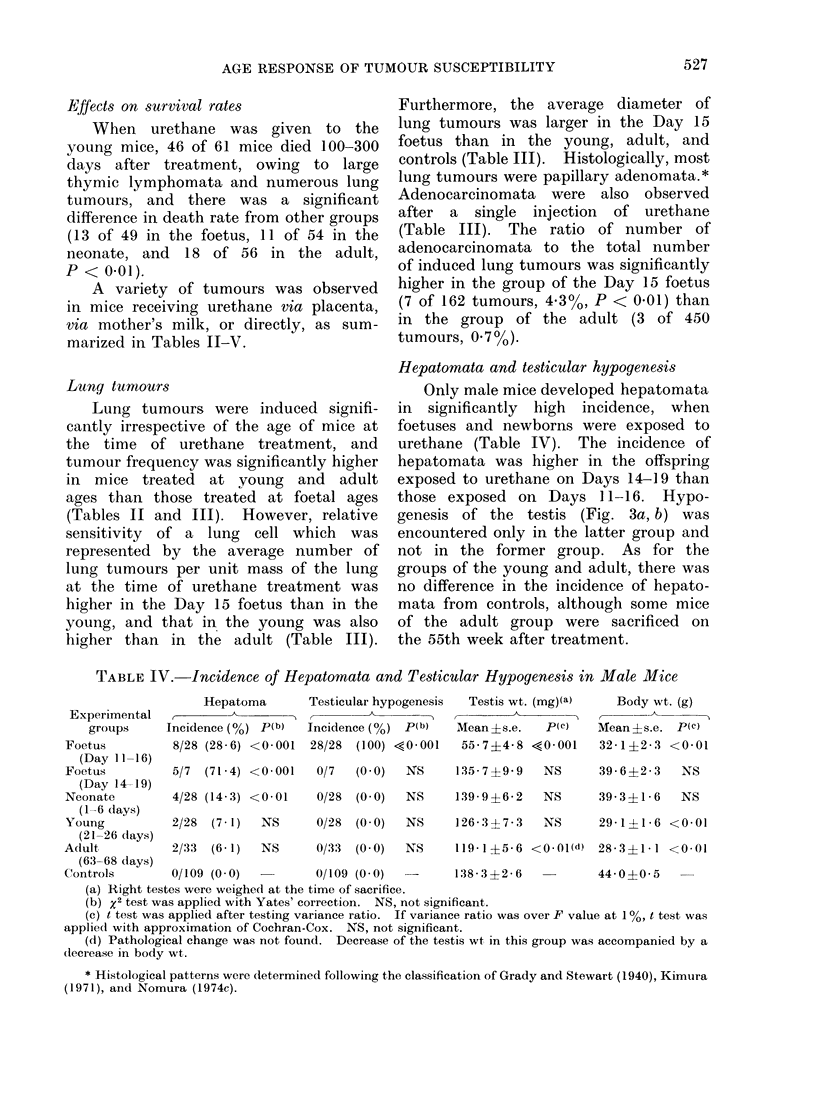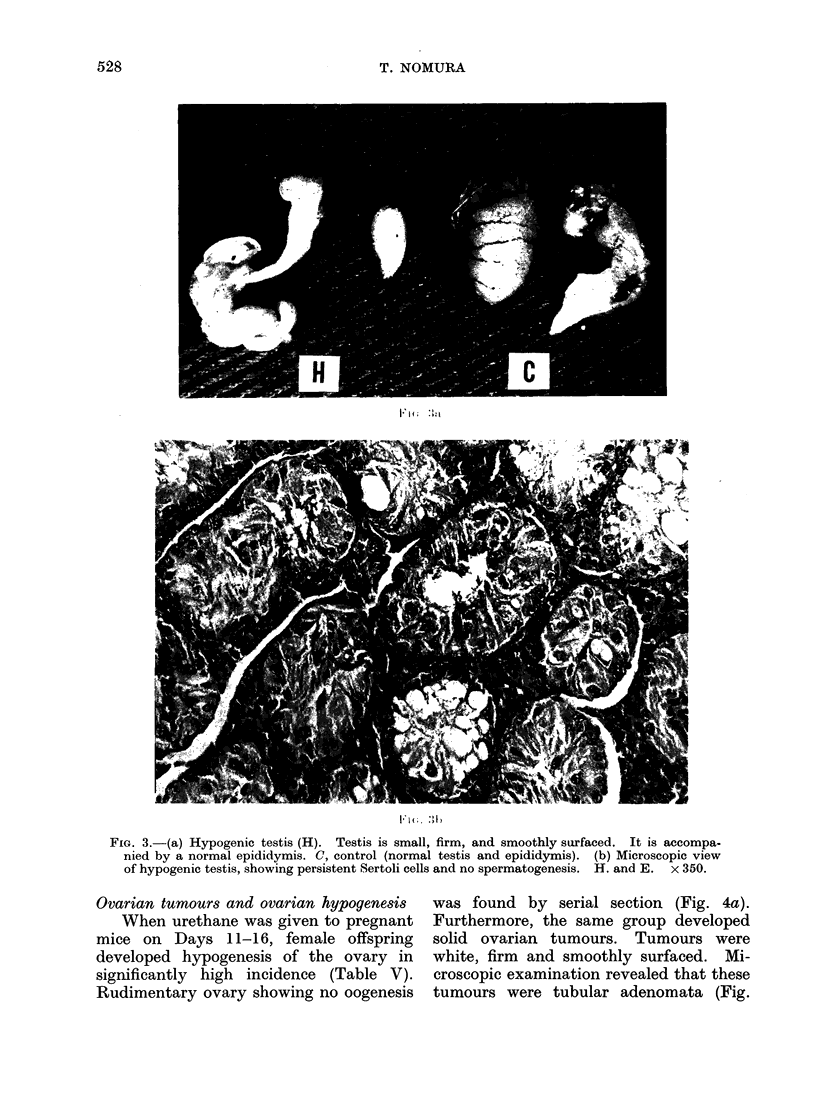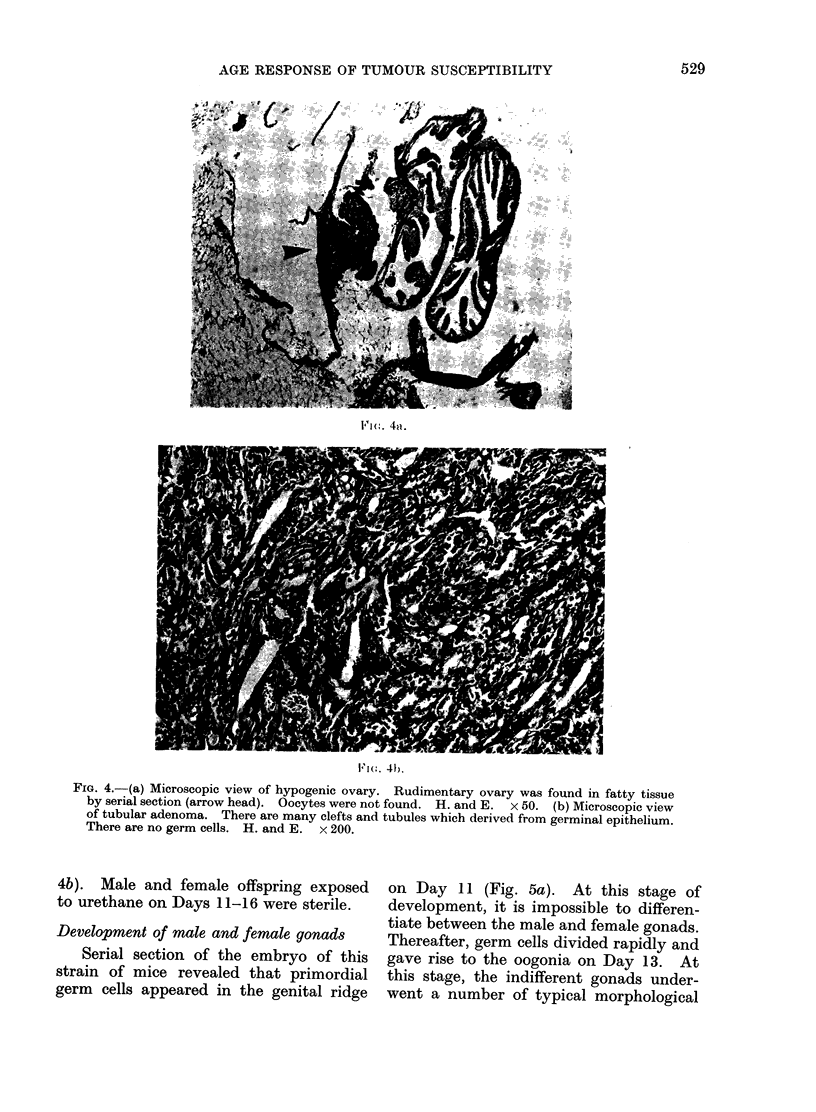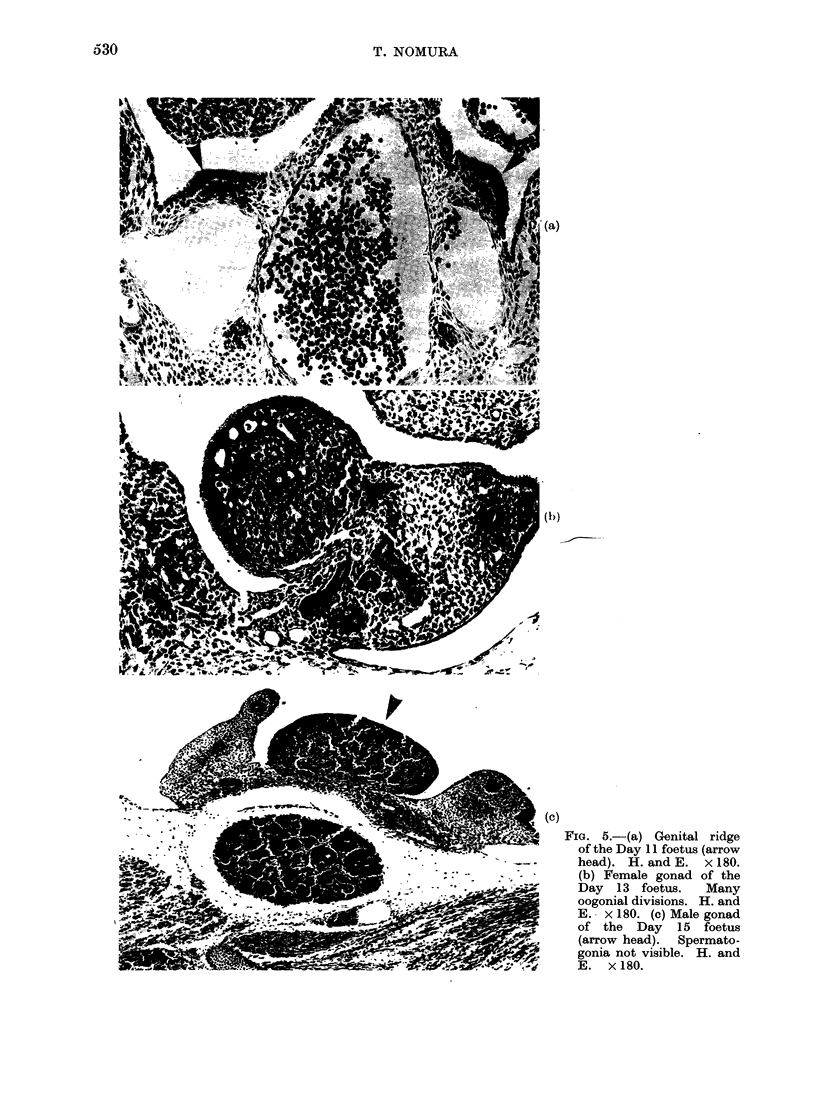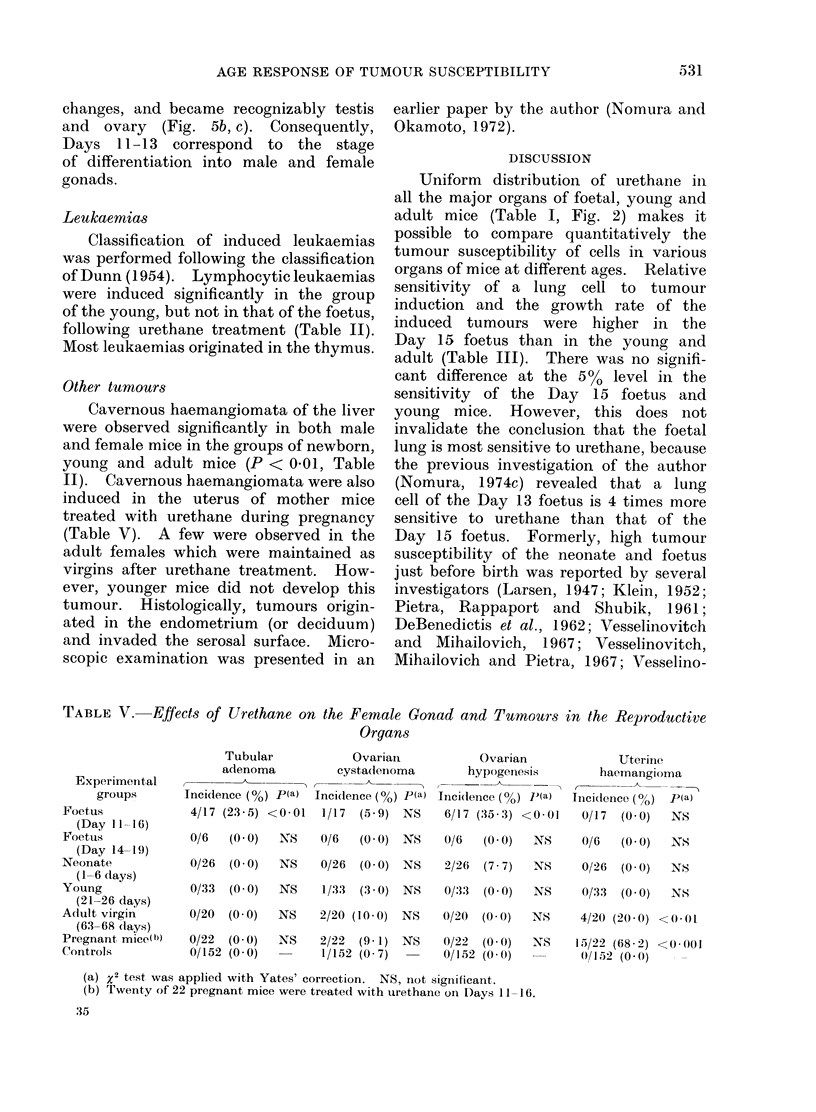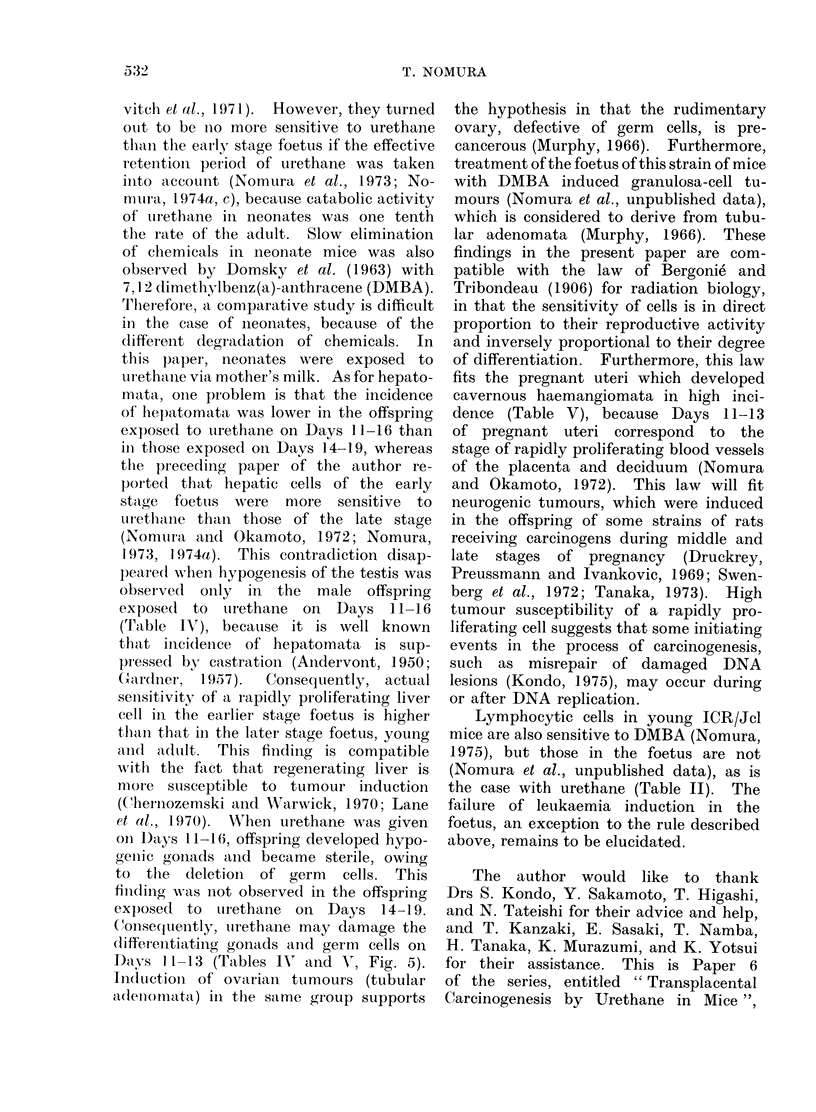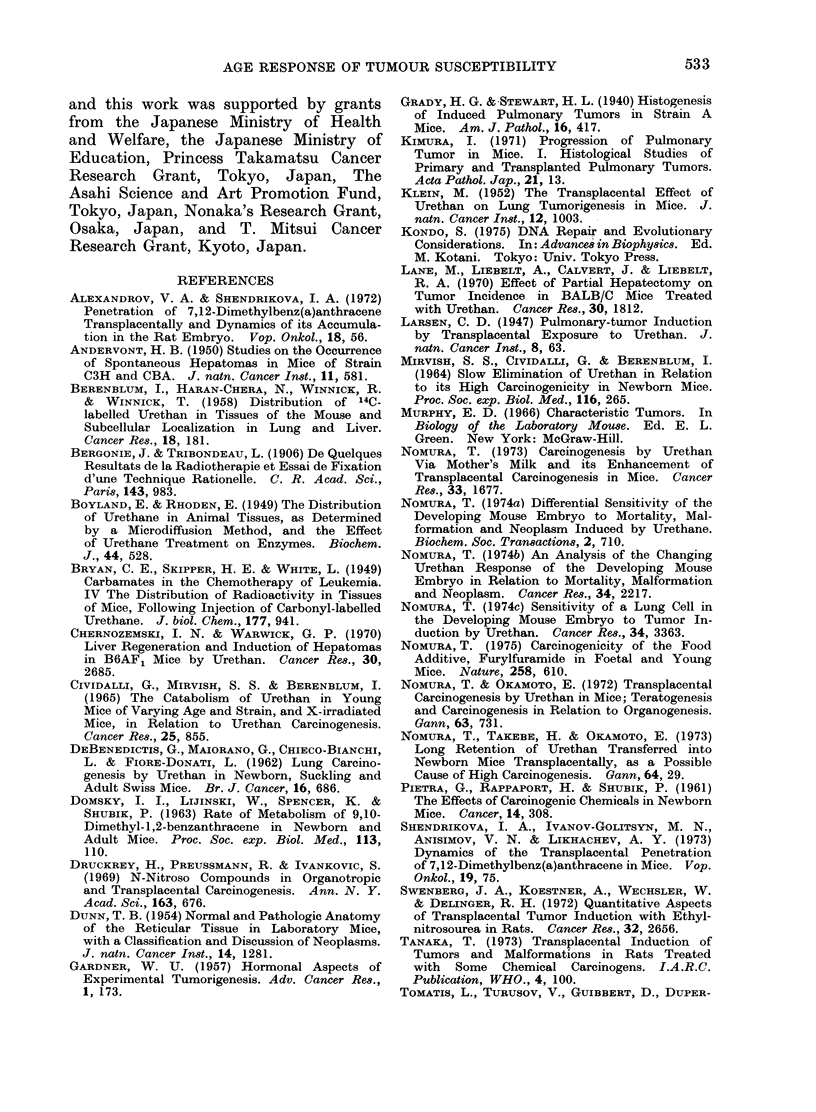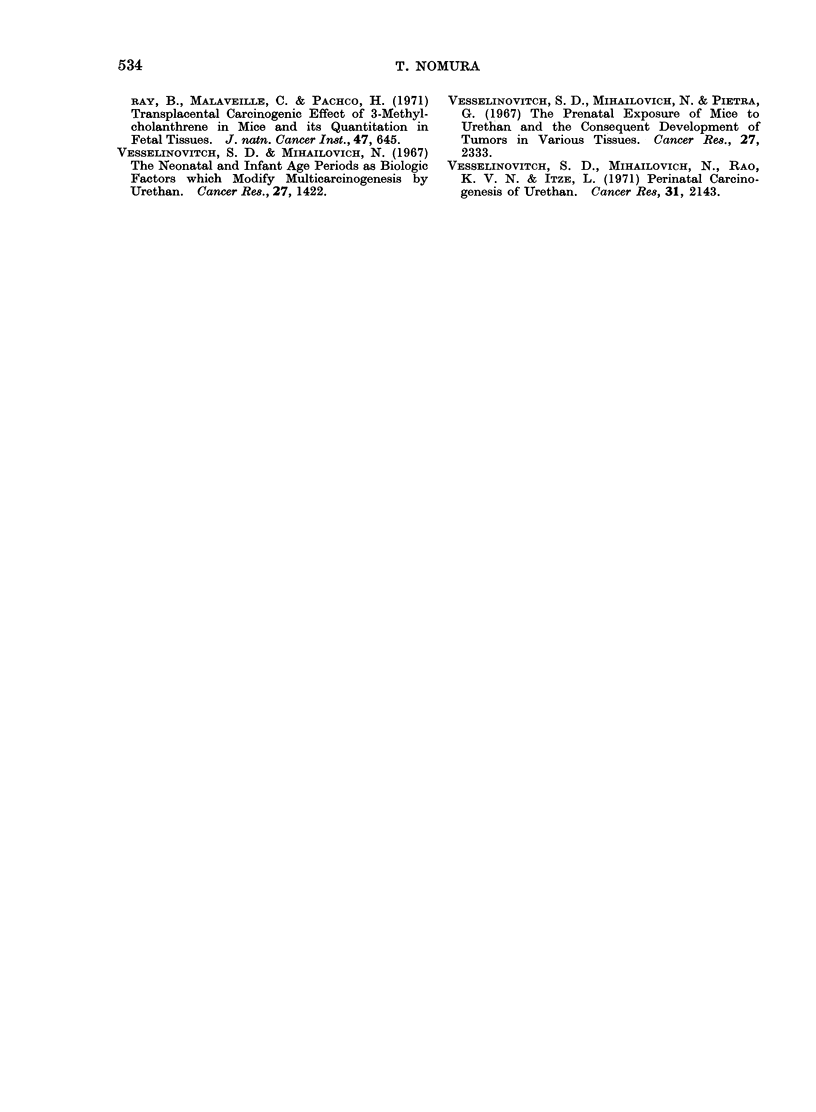# Comparison of tumour susceptibility among various organs of foetal, young and adult ICR/Jcl mice.

**DOI:** 10.1038/bjc.1976.83

**Published:** 1976-05

**Authors:** T. Nomura

## Abstract

**Images:**


*Br. J. Cancer* (1976) **33**, 521

### COMPARISON OF TUMOUR SUSCEPTIBILITY AMONG VARIOUS ORGANS OF FOETAL, YOUNG AND ADULT ICR/Jcl MICE

T. NOMURA*

*From the First Department of Surgery and Institute for Cancer Research, Osaka University Medical School, Fukushima-ku, Osaka 553, Japan*

Received 16 October 1975 Accepted 5 January 1976

**Summary.—Urethane was found to be uniformly distributed in all the major organs of foetal, young and adult ICR/Jcl mice and then to disappear rapidly as measured by the incorporation of urethane-carbonyl-$^{14}C$, thus permitting the accurate comparison of tumour susceptibility of cells in various organs of mice at different ages. Lung tumour frequency (tumours/lung) was significantly higher in mice treated with urethane when young (21 days old) and adult (63 days old) than in those treated *in utero* (Days 11–19 of gestation). When relative sensitivity of a lung cell was calculated as the ratio of average number of tumours per lung per mg of lung at the time of treatment, however, a lung cell of the foetus was more sensitive to urethane than that of the young and adult. Hepatomata were induced significantly only when male foetuses and neonates were exposed to urethane. The offspring exposed to urethane on Days 11–16, however, developed hepatomata in lower incidence than those exposed on Days 14–19, whereas the previous investigation by the author revealed that Days 11–13 correspond to the stage most sensitive to hepatocarcinogenesis. This contradiction was due to the occurrence of testicular hypogenesis (chemical castration) in all offspring of the former group. Differentiating female gonad and rapidly proliferating blood vessels of the placenta and deciduum were also sensitive to tumour induction by urethane. Thus, high tumour susceptibility of rapidly proliferating and undifferentiated cells suggests that some initiating events in the process of carcinogenesis may occur during or after DNA replication. Leukaemia induction in the young mice, but not in the foetus, remains to be elucidated.**

IT HAS BEEN reported by many investigators that a variety of tumours was induced in different strains of mice receiving different carcinogens at foetal, neonatal, young, or adult ages. In order to compare the sensitivity of a cell in an organ of mice at different ages, however, it is of utmost importance to know the dose of the chemical actually reaching the organ and also to know the difference between mice similarly treated at different ages. Urethane possesses the following characteristics: it distributes immediately and uniformly in all organs of young (Cividalli, Mirvish and Berenblum, 1965) and adult mice (Boyland and Rhoden, 1949; Bryan, Skipper and White, 1949; Berenblum *et al.*, 1958) when given parenterally. Furthermore, the author found that urethane, unlike other carcinogens (Tomatis *et al.*, 1971; Alexandrov and Shendrikova, 1972; Shendrikova *et al.*, 1973), can pass through the placenta freely at any stage of pregnancy (Nomura, Takebe and Okamoto, 1973), thus facilitating accurate timing of foetal disturbances and accurate calculation of doses actually reaching the foetus, as in the case of X-rays. Utilizing these unique characteristics of urethane, it was demonstrated that tumour sensitivity of a lung cell in the developing mouse embryo was inversely proportional to the degree of differentiation (Nomura, 1974*a*, *c*). In

* Present address; Department of Medical Genetics, Laboratory of Genetics, University of Wisconsin, Madison, Wisconsin 53706, U.S.A.

the present paper, the work is extended to young and adult mice, and a quantitative analysis of the changing age response of cells in various organs concerning tumour susceptibility is carried out, besides confirming the uniform distribution of urethane in various organs and tissues of foetal, young, and adult ICR/Jcl mice.

### MATERIALS AND METHODS

*Animals.*—ICR/Jcl mice (Japan Central Laboratory for Experimental Animals, Tokyo Japan) were used. An oestrous female was placed in a cage with a breeder male at 10.00 pm, and next morning a vaginal plug was sought to determine Day 1 of gestation. Details have been reported previously (Nomura and Okamoto, 1972).

*Urethane.*—White crystalline ethyl carbamate (Wako Pure Chemical Ind., Ltd., Osaka, Japan), m.p. 48–51 °C, was used. Solutions of 5 and 15% urethane in distilled water were prepared just before use.

*Measurement of organ weight.*—Pregnant mice were sacrificed on Days 11, 12, 13, 14, 15, 16, 17, or 19 of gestation, and their foetuses were weighed. After fixation in 20% neutral formaldehyde solution, brain, thymus, lung, liver, and right kidney were weighed, and submitted to microscopic examinations by serial section. Groups of 10 male and female mice were sacrificed at birth, and on the 1st, 2nd, 3rd, 4th, 6th, 7th, 14th, 21st, 28th, 35th, 42nd, 49th, 56th, 63rd, and 70th days after birth, and their organs (brain, thymus, lung, liver, spleen, right kidney, right testis, and right ovary) were weighed.

*Radioactivity of $^{14}C$-labelled urethane.*—Urethane-carbonyl-$^{14}C$, 0·2 $\mu$Ci (0·568 $\mu$g)/g body weight (sp. act., 31·4 mCi/mmol, Department des Radioelements, France) was injected s.c. into newborns (within 12 h of birth), young females (21 days old), adult virgin females (65 days old), pregnant mice (on Day 15), and lactating mother mice (within 12 h post partum). These mice were sacrificed 1, 2, 4, and 6 h after injection, and alkali-labile $^{14}CO_2$ in the blood, lung, liver, brain and uterus (placenta) of the young, adult virgin female, pregnant mice and their foetuses was measured by liquid scintillation counter following the application of the method reported by Cividalli *et al.* (1965). For newborns and sucklings, radioactivity was measured 2, 6, 12, 24 and 48 h after injection.

*Treatment during pregnancy.*—Thirty-one pregnant mice received 6 subcutaneous injections of urethane (0·5 mg/g body wt) once a day on Days 11–16 or on Days 14–19. Their offspring were foster-nursed by untreated lactating mothers immediately after birth. In order to study the relative sensitivity of a lung cell for tumour induction, 9 pregnant mice received a single injection of urethane (1·5 mg/g body wt) on Day 15. Offspring were separated from mothers 4 weeks after birth, and females were maintained as virgins.

*Treatment during lactation.*—Ten lactating mice received 6 subcutaneous injections of urethane (0·5 mg/g body wt) on the 1st, 2nd, 3rd, 4th, 5th and 6th days post partum. The first treatment was given 12 h after parturition. Offspring were nursed by these urethane-treated lactating mice, and separated from mothers 4 weeks after birth.

*Treatment of young and adult mice.*—Both male and female (virgin) mice received 6 s.c. injections of urethane (0·5 mg/g body wt) daily on the 21st to 26th days (young group) or on the 63rd to 68th days (adult group) after birth. Female mice also received a single injection of urethane at a dose of 1·5 mg/g body wt on the 21st or 64th day after birth. These urethane-treated mice were maintained on mouse diet CA-1 (Nomura, 1974*b*), and sacrificed in the 40th–43rd week after treatment. The mice which died later than 30 weeks after urethane treatment were included in the effective number of animals because considerable numbers of mice in the young group died before sacrifice. For the experiment carried out to estimate tumour sensitivity of a lung cell, all animals were sacrificed on the 32nd week after treatment. Gross pathological lesions were examined for tumours and specimens were submitted to microscopic examination. Lung tumours were counted again after fixation in 20% neutral formaldehyde solution (Nomura and Okamoto, 1972; Nomura, 1974*c*), and diameter of lung tumours was measured by a slide caliper. Relative sensitivity of a lung cell for tumour induction was calculated as the ratio of the average number of tumours per lung to the weight in mg of lung in the concurrent control group at the time of urethane exposure (Nomura, 1974*a*, *c*). The weight of lung was used as a measure of the total number of lung cells (Nomura, 1974*c*).

### RESULTS

### *Growth of ICR/Jcl mice*

Growth of these falls into 4 stages: *i.e.* foetal, neonatal, young and adult stages. All foetal organs grew rapidly until Day 17. Thereafter, growth rate decreased, and physiological weight loss was observed at birth. From 1–5 weeks of age, weight of whole body, lung, liver, kidney, testis and ovary increased exponentially (*young stage*), and thereafter the growth rate of these organs reached a stationary phase (*adult stage*). Reproductive activity of male and female mice appeared at 5–6 weeks. Thymus and spleen grew exponentially until 4 weeks of age. Their weight reached maximum at 5 weeks, and decreased thereafter. Brain weight reached its maximum at 7 days of age, and remained at that level (Fig. 1).

### *Distribution of $^{14}C$-labelled urethane*

When urethane-carbonyl-$^{14}C$ was injected into the young, adult and pregnant mice, there was no difference in the level and half-life of urethane among all organs of the foetus, young, adult female (virgin), and pregnant mice (Fig. 2, Table I). In the case of neonates, however, catabolic activity was approximately one tenth the rate of the young and adult (Table I), as is the case reported by Mirvish, Cividalli and Berenblum (1964). About 2–3% of the maternal concentration of urethane was detected in various organs of sucklings which were nursed by urethane-treated lactating mothers (Fig. 2).

FIG. 1.—Growth of various organs of ICR/Jcl mice. Each point shows the average of 10 organs (Mean ± s.e.). Details of organ weight measurement are given in " Materials and Methods ". Data of the foetus and neonate had been reported previously (Nomura, 1974*b*). ● whole body, ▲ brain, ■ thymus, ○ lung, △ liver, ⊟ spleen, □ kidney, ⊖ testis or ovary.

FIG. 2.—Labelling of urethane-carbonyl-$^{14}C$ in the ICR/Jcl mouse foetus, neonate, suckling, young, adult virgin, and pregnant mice. Urethane-carbonyl-$^{14}C$, 0·2 $\mu$Ci/g body wt, was given s.c. to pregnant mice, lactating mice, neonates, young, and adult virgin mice. Alkali-labile $^{14}CO_2$ (d/min/mg wet tissue) was measured in various organs of the foetus (●), neonate (▲), suckling (□), young (△), adult virgin (■), and pregnant mice (○). Sucklings were exposed to $^{14}C$-labelled urethane *via* mother's milk following urethane treatment to lactating mice. In the case of the foetus, radioactivity of placenta was measured instead of uterus. Details are given in " Materials and Methods ". These decomposition curves of the pregnant mice and their foetuses are approximately equal to those reported in the preceding paper of the author (Nomura *et al.*, 1973), in which radioactivity was measured by total-$^{14}C$.

TABLE I.—*Rate of Loss of Urethane-carbonyl-$^{14}C$ in Various Tissues of the Foetus, Neonate, Young, Adult Virgin and Pregnant Mice*

| | Average half-life (h) in 3 experiments | | | | |
|---|---|---|---|---|---|
| Experimental groups | Blood | Lung | Liver | Brain | Uterus |
| Foetus (Day 15) | 1·7 | 1·7 | 1·8 | 1·7 | 1·8* |
| Newborn (within 12 h) | 17·8 | 17·0 | 17·0 | 18·0 | 17·5 |
| Young (21 days) | 2·0 | 1·9 | 1·9 | 1·9 | 1·9 |
| Adult virgin (65 days) | 1·6 | 1·6 | 1·5 | 1·5 | 1·7 |
| Pregnant mice | 2·0 | 1·8 | 1·7 | 1·6 | 1·8 |

* In the case of the foetus, radioactivity of the placenta was measured instead of uterus.

*Effects on survival rates*

When urethane was given to the young mice, 46 of 61 mice died 100–300 days after treatment, owing to large thymic lymphomata and numerous lung tumours, and there was a significant difference in death rate from other groups (13 of 49 in the foetus, 11 of 54 in the neonate, and 18 of 56 in the adult, $P < 0.01$).

A variety of tumours was observed in mice receiving urethane *via* placenta, *via* mother's milk, or directly, as summarized in Tables II–V.

*Lung tumours*

Lung tumours were induced significantly irrespective of the age of mice at the time of urethane treatment, and tumour frequency was significantly higher in mice treated at young and adult ages than those treated at foetal ages (Tables II and III). However, relative sensitivity of a lung cell which was represented by the average number of lung tumours per unit mass of the lung at the time of urethane treatment was higher in the Day 15 foetus than in the young, and that in the young was also higher than in the adult (Table III). Furthermore, the average diameter of lung tumours was larger in the Day 15 foetus than in the young, adult, and controls (Table III). Histologically, most lung tumours were papillary adenomata.* Adenocarcinomata were also observed after a single injection of urethane (Table III). The ratio of number of adenocarcinomata to the total number of induced lung tumours was significantly higher in the group of the Day 15 foetus (7 of 162 tumours, 4·3%, $P < 0.01$) than in the group of the adult (3 of 450 tumours, 0·7%).

*Hepatomata and testicular hypogenesis*

Only male mice developed hepatomata in significantly high incidence, when foetuses and newborns were exposed to urethane (Table IV). The incidence of hepatomata was higher in the offspring exposed to urethane on Days 14–19 than those exposed on Days 11–16. Hypogenesis of the testis (Fig. 3*a*, *b*) was encountered only in the latter group and not in the former group. As for the groups of the young and adult, there was no difference in the incidence of hepatomata from controls, although some mice of the adult group were sacrificed on the 55th week after treatment.

TABLE IV.—*Incidence of Hepatomata and Testicular Hypogenesis in Male Mice*

| Experimental groups | Hepatoma | | | Testicular hypogenesis | | | Testis wt. (mg)[a] | | Body wt. (g) | |
|---|---|---|---|---|---|---|---|---|---|---|
| | Incidence | (%) | $P^{(b)}$ | Incidence | (%) | $P^{(b)}$ | Mean ± s.e. | $P^{(c)}$ | Mean ± s.e. | $P^{(c)}$ |
| Foetus (Day 11–16) | 8/28 | (28·6) | <0·001 | 28/28 | (100) | ⩽0·001 | 55·7±4·8 | ⩽0·001 | 32·1±2·3 | <0·01 |
| Foetus (Day 14–19) | 5/7 | (71·4) | <0·001 | 0/7 | (0·0) | NS | 135·7±9·9 | NS | 39·6±2·3 | NS |
| Neonate (1–6 days) | 4/28 | (14·3) | <0·01 | 0/28 | (0·0) | NS | 139·9±6·2 | NS | 39·3±1·6 | NS |
| Young (21–26 days) | 2/28 | (7·1) | NS | 0/28 | (0·0) | NS | 126·3±7·3 | NS | 29·1±1·6 | <0·01 |
| Adult (63–68 days) | 2/33 | (6·1) | NS | 0/33 | (0·0) | NS | 119·1±5·6 | <0·01[d] | 28·3±1·1 | <0·01 |
| Controls | 0/109 | (0·0) | — | 0/109 | (0·0) | — | 138·3±2·6 | — | 44·0±0·5 | — |

(a) Right testes were weighed at the time of sacrifice.
(b) $\chi^2$ test was applied with Yates' correction. NS, not significant.
(c) *t* test was applied after testing variance ratio. If variance ratio was over *F* value at 1%, *t* test was applied with approximation of Cochran-Cox. NS, not significant.
(d) Pathological change was not found. Decrease of the testis wt in this group was accompanied by a decrease in body wt.

* Histological patterns were determined following the classification of Grady and Stewart (1940), Kimura (1971), and Nomura (1974*c*).

Fig. 3a.

Fig. 3b.

Fig. 3.—(a) Hypogenic testis (H). Testis is small, firm, and smoothly surfaced. It is accompanied by a normal epididymis. *C*, control (normal testis and epididymis). (b) Microscopic view of hypogenic testis, showing persistent Sertoli cells and no spermatogenesis. H. and E. × 350.

*Ovarian tumours and ovarian hypogenesis*

When urethane was given to pregnant mice on Days 11–16, female offspring developed hypogenesis of the ovary in significantly high incidence (Table V). Rudimentary ovary showing no oogenesis was found by serial section (Fig. 4*a*). Furthermore, the same group developed solid ovarian tumours. Tumours were white, firm and smoothly surfaced. Microscopic examination revealed that these tumours were tubular adenomata (Fig.

FIG. 4a.

FIG. 4b.

FIG. 4.—(a) Microscopic view of hypogenic ovary. Rudimentary ovary was found in fatty tissue by serial section (arrow head). Oocytes were not found. H. and E. ×50. (b) Microscopic view of tubular adenoma. There are many clefts and tubules which derived from germinal epithelium. There are no germ cells. H. and E. ×200.

4*b*). Male and female offspring exposed to urethane on Days 11–16 were sterile.

*Development of male and female gonads*

Serial section of the embryo of this strain of mice revealed that primordial germ cells appeared in the genital ridge on Day 11 (Fig. 5*a*). At this stage of development, it is impossible to differentiate between the male and female gonads. Thereafter, germ cells divided rapidly and gave rise to the oogonia on Day 13. At this stage, the indifferent gonads underwent a number of typical morphological

(a)

(b)

(c)

Fig. 5.—(a) Genital ridge of the Day 11 foetus (arrow head). H. and E. ×180. (b) Female gonad of the Day 13 foetus. Many oogonial divisions. H. and E. ×180. (c) Male gonad of the Day 15 foetus (arrow head). Spermatogonia not visible. H. and E. ×180.

changes, and became recognizably testis and ovary (Fig. 5*b*, *c*). Consequently, Days 11–13 correspond to the stage of differentiation into male and female gonads.

*Leukaemias*

Classification of induced leukaemias was performed following the classification of Dunn (1954). Lymphocytic leukaemias were induced significantly in the group of the young, but not in that of the foetus, following urethane treatment (Table II). Most leukaemias originated in the thymus.

*Other tumours*

Cavernous haemangiomata of the liver were observed significantly in both male and female mice in the groups of newborn, young and adult mice ($P < 0{\cdot}01$, Table II). Cavernous haemangiomata were also induced in the uterus of mother mice treated with urethane during pregnancy (Table V). A few were observed in the adult females which were maintained as virgins after urethane treatment. However, younger mice did not develop this tumour. Histologically, tumours originated in the endometrium (or deciduum) and invaded the serosal surface. Microscopic examination was presented in an earlier paper by the author (Nomura and Okamoto, 1972).

### DISCUSSION

Uniform distribution of urethane in all the major organs of foetal, young and adult mice (Table I, Fig. 2) makes it possible to compare quantitatively the tumour susceptibility of cells in various organs of mice at different ages. Relative sensitivity of a lung cell to tumour induction and the growth rate of the induced tumours were higher in the Day 15 foetus than in the young and adult (Table III). There was no significant difference at the 5% level in the sensitivity of the Day 15 foetus and young mice. However, this does not invalidate the conclusion that the foetal lung is most sensitive to urethane, because the previous investigation of the author (Nomura, 1974*c*) revealed that a lung cell of the Day 13 foetus is 4 times more sensitive to urethane than that of the Day 15 foetus. Formerly, high tumour susceptibility of the neonate and foetus just before birth was reported by several investigators (Larsen, 1947; Klein, 1952; Pietra, Rappaport and Shubik, 1961; DeBenedictis *et al.*, 1962; Vesselinovitch and Mihailovich, 1967; Vesselinovitch, Mihailovich and Pietra, 1967; Vesselino-

TABLE V.—*Effects of Urethane on the Female Gonad and Tumours in the Reproductive Organs*

| Experimental groups | Tubular adenoma | | | Ovarian cystadenoma | | | Ovarian hypogenesis | | | Uterine haemangioma | | |
|---|---|---|---|---|---|---|---|---|---|---|---|---|
| | Incidence | (%) | $P^{(a)}$ | Incidence | (%) | $P^{(a)}$ | Incidence | (%) | $P^{(a)}$ | Incidence | (%) | $P^{(a)}$ |
| Foetus (Day 11–16) | 4/17 | (23·5) | <0·01 | 1/17 | (5·9) | NS | 6/17 | (35·3) | <0·01 | 0/17 | (0·0) | NS |
| Foetus (Day 14–19) | 0/6 | (0·0) | NS | 0/6 | (0·0) | NS | 0/6 | (0·0) | NS | 0/6 | (0·0) | NS |
| Neonate (1–6 days) | 0/26 | (0·0) | NS | 0/26 | (0·0) | NS | 2/26 | (7·7) | NS | 0/26 | (0·0) | NS |
| Young (21–26 days) | 0/33 | (0·0) | NS | 1/33 | (3·0) | NS | 0/33 | (0·0) | NS | 0/33 | (0·0) | NS |
| Adult virgin (63–68 days) | 0/20 | (0·0) | NS | 2/20 | (10·0) | NS | 0/20 | (0·0) | NS | 4/20 | (20·0) | <0·01 |
| Pregnant mice[b] | 0/22 | (0·0) | NS | 2/22 | (9·1) | NS | 0/22 | (0·0) | NS | 15/22 | (68·2) | <0·001 |
| Controls | 0/152 | (0·0) | — | 1/152 | (0·7) | — | 0/152 | (0·0) | — | 0/152 | (0·0) | — |

(a) $\chi^2$ test was applied with Yates' correction. NS, not significant.
(b) Twenty of 22 pregnant mice were treated with urethane on Days 11–16.

vitch *et al.*, 1971). However, they turned out to be no more sensitive to urethane than the early stage foetus if the effective retention period of urethane was taken into account (Nomura *et al.*, 1973; Nomura, 1974*a*, *c*), because catabolic activity of urethane in neonates was one tenth the rate of the adult. Slow elimination of chemicals in neonate mice was also observed by Domsky *et al.* (1963) with 7,12 dimethylbenz(a)-anthracene (DMBA). Therefore, a comparative study is difficult in the case of neonates, because of the different degradation of chemicals. In this paper, neonates were exposed to urethane via mother's milk. As for hepatomata, one problem is that the incidence of hepatomata was lower in the offspring exposed to urethane on Days 11–16 than in those exposed on Days 14–19, whereas the preceding paper of the author reported that hepatic cells of the early stage foetus were more sensitive to urethane than those of the late stage (Nomura and Okamoto, 1972; Nomura, 1973, 1974*a*). This contradiction disappeared when hypogenesis of the testis was observed only in the male offspring exposed to urethane on Days 11–16 (Table IV), because it is well known that incidence of hepatomata is suppressed by castration (Andervont, 1950; Gardner, 1957). Consequently, actual sensitivity of a rapidly proliferating liver cell in the earlier stage foetus is higher than that in the later stage foetus, young and adult. This finding is compatible with the fact that regenerating liver is more susceptible to tumour induction (Chernozemski and Warwick, 1970; Lane *et al.*, 1970). When urethane was given on Days 11–16, offspring developed hypogenic gonads and became sterile, owing to the deletion of germ cells. This finding was not observed in the offspring exposed to urethane on Days 14–19. Consequently, urethane may damage the differentiating gonads and germ cells on Days 11–13 (Tables IV and V, Fig. 5). Induction of ovarian tumours (tubular adenomata) in the same group supports the hypothesis in that the rudimentary ovary, defective of germ cells, is precancerous (Murphy, 1966). Furthermore, treatment of the foetus of this strain of mice with DMBA induced granulosa-cell tumours (Nomura *et al.*, unpublished data), which is considered to derive from tubular adenomata (Murphy, 1966). These findings in the present paper are compatible with the law of Bergonié and Tribondeau (1906) for radiation biology, in that the sensitivity of cells is in direct proportion to their reproductive activity and inversely proportional to their degree of differentiation. Furthermore, this law fits the pregnant uteri which developed cavernous haemangiomata in high incidence (Table V), because Days 11–13 of pregnant uteri correspond to the stage of rapidly proliferating blood vessels of the placenta and deciduum (Nomura and Okamoto, 1972). This law will fit neurogenic tumours, which were induced in the offspring of some strains of rats receiving carcinogens during middle and late stages of pregnancy (Druckrey, Preussmann and Ivankovic, 1969; Swenberg *et al.*, 1972; Tanaka, 1973). High tumour susceptibility of a rapidly proliferating cell suggests that some initiating events in the process of carcinogenesis, such as misrepair of damaged DNA lesions (Kondo, 1975), may occur during or after DNA replication.

Lymphocytic cells in young ICR/Jcl mice are also sensitive to DMBA (Nomura, 1975), but those in the foetus are not (Nomura *et al.*, unpublished data), as is the case with urethane (Table II). The failure of leukaemia induction in the foetus, an exception to the rule described above, remains to be elucidated.

The author would like to thank Drs S. Kondo, Y. Sakamoto, T. Higashi, and N. Tateishi for their advice and help, and T. Kanzaki, E. Sasaki, T. Namba, H. Tanaka, K. Murazumi, and K. Yotsui for their assistance. This is Paper 6 of the series, entitled " Transplacental Carcinogenesis by Urethane in Mice ", and this work was supported by grants from the Japanese Ministry of Health and Welfare, the Japanese Ministry of Education, Princess Takamatsu Cancer Research Grant, Tokyo, Japan, The Asahi Science and Art Promotion Fund, Tokyo, Japan, Nonaka's Research Grant, Osaka, Japan, and T. Mitsui Cancer Research Grant, Kyoto, Japan.

### REFERENCES

ALEXANDROV, V. A. & SHENDRIKOVA, I. A. (1972) Penetration of 7,12-Dimethylbenz(a)anthracene Transplacentally and Dynamics of its Accumulation in the Rat Embryo. *Vop. Onkol.*, **18**, 56.

ANDERVONT, H. B. (1950) Studies on the Occurrence of Spontaneous Hepatomas in Mice of Strain C3H and CBA. *J. natn. Cancer Inst.*, **11**, 581.

BERENBLUM, I., HARAN-CHERA, N., WINNICK, R. & WINNICK, T. (1958) Distribution of $^{14}$C-labelled Urethan in Tissues of the Mouse and Subcellular Localization in Lung and Liver. *Cancer Res.*, **18**, 181.

BERGONIE, J. & TRIBONDEAU, L. (1906) De Quelques Resultats de la Radiotherapie et Essai de Fixation d'une Technique Rationelle. *C. R. Acad. Sci., Paris*, **143**, 983.

BOYLAND, E. & RHODEN, E. (1949) The Distribution of Urethane in Animal Tissues, as Determined by a Microdiffusion Method, and the Effect of Urethane Treatment on Enzymes. *Biochem. J.*, **44**, 528.

BRYAN, C. E., SKIPPER, H. E. & WHITE, L. (1949) Carbamates in the Chemotherapy of Leukemia. IV The Distribution of Radioactivity in Tissues of Mice, Following Injection of Carbonyl-labelled Urethane. *J. biol. Chem.*, **177**, 941.

CHERNOZEMSKI, I. N. & WARWICK, G. P. (1970) Liver Regeneration and Induction of Hepatomas in B6AF$_1$ Mice by Urethan. *Cancer Res.*, **30**, 2685.

CIVIDALLI, G., MIRVISH, S. S. & BERENBLUM, I. (1965) The Catabolism of Urethan in Young Mice of Varying Age and Strain, and X-irradiated Mice, in Relation to Urethan Carcinogenesis. *Cancer Res.*, **25**, 855.

DEBENEDICTIS, G., MAIORANO, G., CHIECO-BIANCHI, L. & FIORE-DONATI, L. (1962) Lung Carcinogenesis by Urethan in Newborn, Suckling and Adult Swiss Mice. *Br. J. Cancer*, **16**, 686.

DOMSKY, I. I., LIJINSKI, W., SPENCER, K. & SHUBIK, P. (1963) Rate of Metabolism of 9,10-Dimethyl-1,2-benzanthracene in Newborn and Adult Mice. *Proc. Soc. exp. Biol. Med.*, **113**, 110.

DRUCKREY, H., PREUSSMANN, R. & IVANKOVIC, S. (1969) N-Nitroso Compounds in Organotropic and Transplacental Carcinogenesis. *Ann. N. Y. Acad. Sci.*, **163**, 676.

DUNN, T. B. (1954) Normal and Pathologic Anatomy of the Reticular Tissue in Laboratory Mice, with a Classification and Discussion of Neoplasms. *J. natn. Cancer Inst.*, **14**, 1281.

GARDNER, W. U. (1957) Hormonal Aspects of Experimental Tumorigenesis. *Adv. Cancer Res.*, **1**, 173.

GRADY, H. G. & STEWART, H. L. (1940) Histogenesis of Induced Pulmonary Tumors in Strain A Mice. *Am. J. Pathol.*, **16**, 417.

KIMURA, I. (1971) Progression of Pulmonary Tumor in Mice. I. Histological Studies of Primary and Transplanted Pulmonary Tumors. *Acta Pathol. Jap.*, **21**, 13.

KLEIN, M. (1952) The Transplacental Effect of Urethan on Lung Tumorigenesis in Mice. *J. natn. Cancer Inst.*, **12**, 1003.

KONDO, S. (1975) DNA Repair and Evolutionary Considerations. In: *Advances in Biophysics*. Ed. M. Kotani. Tokyo: Univ. Tokyo Press.

LANE, M., LIEBELT, A., CALVERT, J. & LIEBELT, R. A. (1970) Effect of Partial Hepatectomy on Tumor Incidence in BALB/C Mice Treated with Urethan. *Cancer Res.*, **30**, 1812.

LARSEN, C. D. (1947) Pulmonary-tumor Induction by Transplacental Exposure to Urethan. *J. natn. Cancer Inst.*, **8**, 63.

MIRVISH, S. S., CIVIDALLI, G. & BERENBLUM, I. (1964) Slow Elimination of Urethan in Relation to its High Carcinogenicity in Newborn Mice. *Proc. Soc. exp. Biol. Med.*, **116**, 265.

MURPHY, E. D. (1966) Characteristic Tumors. In *Biology of the Laboratory Mouse*. Ed. E. L. Green. New York: McGraw-Hill.

NOMURA, T. (1973) Carcinogenesis by Urethan Via Mother's Milk and its Enhancement of Transplacental Carcinogenesis in Mice. *Cancer Res.*, **33**, 1677.

NOMURA, T. (1974*a*) Differential Sensitivity of the Developing Mouse Embryo to Mortality, Malformation and Neoplasm Induced by Urethane. *Biochem. Soc. Transactions*, **2**, 710.

NOMURA, T. (1974*b*) An Analysis of the Changing Urethan Response of the Developing Mouse Embryo in Relation to Mortality, Malformation and Neoplasm. *Cancer Res.*, **34**, 2217.

NOMURA, T. (1974*c*) Sensitivity of a Lung Cell in the Developing Mouse Embryo to Tumor Induction by Urethan. *Cancer Res.*, **34**, 3363.

NOMURA, T. (1975) Carcinogenicity of the Food Additive, Furylfuramide in Foetal and Young Mice. *Nature*, **258**, 610.

NOMURA, T. & OKAMOTO, E. (1972) Transplacental Carcinogenesis by Urethan in Mice; Teratogenesis and Carcinogenesis in Relation to Organogenesis. *Gann*, **63**, 731.

NOMURA, T., TAKEBE, H. & OKAMOTO, E. (1973) Long Retention of Urethan Transferred into Newborn Mice Transplacentally, as a Possible Cause of High Carcinogenesis. *Gann*, **64**, 29.

PIETRA, G., RAPPAPORT, H. & SHUBIK, P. (1961) The Effects of Carcinogenic Chemicals in Newborn Mice. *Cancer*, **14**, 308.

SHENDRIKOVA, I. A., IVANOV-GOLITSYN, M. N., ANISIMOV, V. N. & LIKHACHEV, A. Y. (1973) Dynamics of the Transplacental Penetration of 7,12-Dimethylbenz(a)anthracene in Mice. *Vop. Onkol.*, **19**, 75.

SWENBERG, J. A., KOESTNER, A., WECHSLER, W. & DELINGER, R. H. (1972) Quantitative Aspects of Transplacental Tumor Induction with Ethylnitrosourea in Rats. *Cancer Res.*, **32**, 2656.

TANAKA, T. (1973) Transplacental Induction of Tumors and Malformations in Rats Treated with Some Chemical Carcinogens. *I.A.R.C. Publication, WHO.*, **4**, 100.

TOMATIS, L., TURUSOV, V., GUIBBERT, D., DUPER-

RAY, B., MALAVEILLE, C. & PACHCO, H. (1971) Transplacental Carcinogenic Effect of 3-Methylcholanthrene in Mice and its Quantitation in Fetal Tissues. *J. natn. Cancer Inst.*, **47**, 645.

VESSELINOVITCH, S. D. & MIHAILOVICH, N. (1967) The Neonatal and Infant Age Periods as Biologic Factors which Modify Multicarcinogenesis by Urethan. *Cancer Res.*, **27**, 1422.

VESSELINOVITCH, S. D., MIHAILOVICH, N. & PIETRA, G. (1967) The Prenatal Exposure of Mice to Urethan and the Consequent Development of Tumors in Various Tissues. *Cancer Res.*, **27**, 2333.

VESSELINOVITCH, S. D., MIHAILOVICH, N., RAO, K. V. N. & ITZE, L. (1971) Perinatal Carcinogenesis of Urethan. *Cancer Res*, **31**, 2143.